# A New Multiconstraint Method for Determining the Optimal Cable Stresses in Cable-Stayed Bridges

**DOI:** 10.1155/2014/503016

**Published:** 2014-06-22

**Authors:** B. Asgari, S. A. Osman, A. Adnan

**Affiliations:** ^1^Department of Civil & Structural Engineering, Faculty of Engineering & Built Environment, Universiti Kebangsaan Malaysia (UKM), 43600 Bangi, Selangor, Malaysia; ^2^Faculty of Civil Engineering, Universiti Teknologi Malaysia (UTM), 81310 Skudai, Johor, Malaysia

## Abstract

Cable-stayed bridges are one of the most popular types of long-span bridges. The structural behaviour of cable-stayed bridges is sensitive to the load distribution between the girder, pylons, and cables. The determination of pretensioning cable stresses is critical in the cable-stayed bridge design procedure. By finding the optimum stresses in cables, the load and moment distribution of the bridge can be improved. In recent years, different research works have studied iterative and modern methods to find optimum stresses of cables. However, most of the proposed methods have limitations in optimising the structural performance of cable-stayed bridges. This paper presents a multiconstraint optimisation method to specify the optimum cable forces in cable-stayed bridges. The proposed optimisation method produces less bending moments and stresses in the bridge members and requires shorter simulation time than other proposed methods. The results of comparative study show that the proposed method is more successful in restricting the deck and pylon displacements and providing uniform deck moment distribution than unit load method (ULM). The final design of cable-stayed bridges can be optimised considerably through proposed multiconstraint optimisation method.

## 1. Introduction

### 1.1. Background

The construction of cable-stayed bridges is rapidly increasing all over the world. The structural behaviour of cable-stayed bridges is significantly sensitive to the load distribution between the girder, pylons, and cables. Cables are one of the main parts of a cable-stayed bridge. They transfer the dead load and traffic load of the girders to the pylons. They are also sensitive to dynamic loads such as wind loads, which make their health monitoring a serious issue [1–4]. Determining the optimum distribution of tensioning forces of stay cables is an important step in the design process of cable-stayed bridge, which plays a major role in the construction of cable-stayed bridges. With pretensioning each cable, more stability and less simulation time will be achieved. Consequently, finding the tensile stresses in cables is critical in cost effective design of cable-stayed bridges.

The structural complexity of cable-stayed bridges makes their design process a serious engineering concern. Lazar et al. [5] and Tori et al. [6] were among the first to study the optimisation of cable force in cable-stayed bridges. In recent years, different iterative methods have been proposed to improve the performance of cable-stayed bridges through optimisation of cable forces. Wang et al. [7, 8] suggested a shape-finding procedure (zero displacement method) based on the dead load of the girders and pylons in which cable sag nonlinearity was included. A two-loop iteration method for shape finding was performed using the equilibrium iteration and shape iteration loops. Recent investigations on the proposed method reveal that this method has problem in convergence for long-span cable-stayed bridges which is a gap in the aforementioned method. Moreover, there may be unbalanced horizontal forces on the pylon in asymmetric cable-stayed bridges that will induce large bending moments and deflections.

Chen et al. [9] proposed a force equilibrium method for finding the cable stresses in cable-stayed bridges. They considered three stages of the structure model in optimisation procedure. Instead of the displacement constraints, the bending moments are considered controlling parameters in this study. Because the method only works on the equilibrium of forces, nonlinearities are not considered when defining the initial cable forces. This approach is more time-consuming than the other methods because of the three modelling stages of the analysis. Janjic et al. [10] presented the unit load method (ULM) for finding the cable stresses in cable-stayed bridges based on the idea of Bruer et al. [11]. In the ULM method the adequate factors should be multiplied to the applied unit loads based on certain constraints, such as deflections. Recently, Lee et al. [12], Lonetti and Pascuzzo [13], and Zhang and Wu [14] also applied iterative methods to optimise the tensioning strategy for cable-stayed bridges and its effect on the construction process.

Moreover, in recent years, variety of research works have applied modern methods for finding the cable forces in cable-stayed bridges [15–19]. A scheme based on genetic algorithm (GA) is applied for optimising the cable-stayed bridges by Lute et al. [15]. The computation time of the genetic algorithm has been reduced employing a support vector machine. However, the effect of posttensioning cable forces has not been considered in this optimisation scheme. More recently, Baldomir et al. [16] investigated cable optimisation of long span cable-stayed bridges. The cross-section of stay cables as well as constraint of cable stress and deck displacement is considered in the optimisation strategy. Hassan et al. [17, 18] and Hassan [19] presented a new technique for optimal design of cable-stayed bridges with semi-fan arrangements based on GA. The presented modern methods demand large number of complex variables.

### 1.2. Problem Statement

In cable-stayed bridges, as the number of stay cables is significant, the number of design variables could be quite large and this can lead to potential numerical problems. The modern complex models with a large number of variables do not furnish an applicable method to find cable forces for engineering simulations and there is a need to introduce a more simplified accurate model to find the optimised cable forces for cable-stayed bridges.

Certain numbers of the recent proposed optimisation methods use an iterative process to determine the economical solution. Most of the proposed optimisation approaches have limitations in determining the optimal pretensioning forces. Among proposed iterative optimization strategies, the ULM method seems to be a prominent fundamental method for optimising the cable forces considering its easy extensibility and applicability to real construction engineering problems. The main advantage of ULM is that it can provide a tensioning strategy during the individual construction stages by taking time-dependent effects and geometrically nonlinear behaviour into account. Lee et al. [12] proposed an iterative method based on ULM which adds another constraint in the optimisation process. However, the recent studies on the girder moment distribution of cable-stayed bridges show that ULM can be further improved because it does not produce well-distributed cable forces and girder moments in long-span cable-stayed bridges.

The main purpose of this paper is to present an effective multiconstraint optimisation strategy for cable-stayed bridges based on the application of an inverse problem through ULM method resulting in reduced stresses and shorter simulation times than the other proposed approaches. Adjusting local optimum values of cable stresses is also possible through the proposed optimization technique.

The design data of Japan's Tatara bridge is considered in this analytical study through ANSYS (12) [20].

### 1.3. Objectives of the Research

A new multiconstraint optimisation method to obtain posttensioning cable forces for cable-stayed bridges is employed in this research work. The main objectives of this study are to determine the optimum posttensioning cable forces for cable-stayed bridges through multiconstraint optimisation method under the action of dead load and to compare the posttensioning cable forces from the ULM method and the proposed multiconstraint method.

## 2. Multiconstraint Iterative Method

A multiconstraint optimisation method is proposed in this study for finding the cable stresses in long-span cable-stayed bridges. Under the combination of the dead load and cable forces, the first estimation of pretensioning cable forces is taken based on the ULM because the computations are less difficult to converge compared to the other iterative methods. Multiconstraints are applied to make the moment distribution along the deck uniform and minimise the member stresses and also deflections.

**Figure 1:**
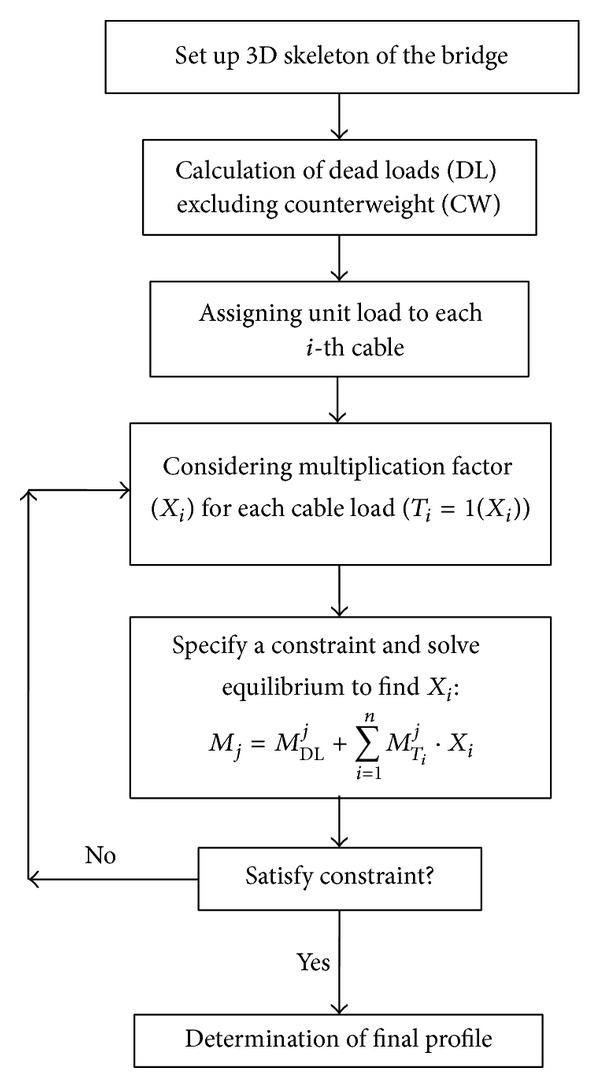
Scheme of ULM method.

To better understand the applied method for optimising cable forces, the scheme of ULM method is presented in Figure 1.

The ULM defines the moment distribution in the structure (*M*_*j*_) for* j* points along the main girder as the sum of the moments created under the action of dead load (*M*_DL_^*j*^) and also unit cable forces (*M*_*T*_*i*__^*j*^), which is multiplied by an unknown factor (*X*_*i*_). The structure will be analysed by adjusting constraints to achieve the pretensioning forces (*T*_*i*_) in the cables. The constraints used by Janjic et al. [10] to find the optimum cable forces are as follows:

- there should be no out-of-plane displacement at the top of pylons;
- there should be no vertical displacement of the deck under dead load.

However, the ULM does not produce well-distributed cable forces and girder moments in long-span cable-stayed bridges [12]. Cable pretensions are difficult to establish due to the limited constraints used to optimise the cable forces. The multiconstraint method adds more constrains to the ULM optimisation strategy to make the moment distribution along the deck uniform and minimise the member stresses.

The following constraints are applied in the proposed method based on ULM to obtain a uniform bending moment distribution.

1. The pylon should be kept straight vertically, which means that the resultant horizontal components of the cable forces will reach equilibrium with almost no out-of-plane displacement produced at the top point of the pylons. To avoid convergence problems in the iterative procedure, the upper and lower bound of 2 × 10^−2^ m are considered to satisfy this constraint  (*δ*_*t*_ < *δ*, *δ* = 2 × 10^−2^ m).
2. The vertical displacement of the girder should be kept at approximately zero because the initial configuration of the bridge should be maintained after pretensioning and before applying the live load. To avoid convergence problems in the iterative procedure, the upper and lower bound of 2 × 10^−2^ m are also considered to satisfy this constraint (*δ*_*d*_ < *δ*, *δ* = 2 × 10^−2^ m). This constraint should be satisfied in each control point (*j*) along the main girder. It is predictable that the middle of the main span would be one of the critical locations for satisfying this criterion.

To produce optimal cable tensions, the following constraints are additionally proposed in this study through an iterative method.

(3) There should be no axial force in the middle of the main span, meaning that the cable forces should not produce extra forces in the girder (*N* = 0).
(4) Relaxation is significantly accelerated in steel cables when the permanent stress is greater than 50% of the tensile strength. Therefore, the stresses from permanent loads should not exceed 0.45 of the tensile strength [21]. Each obtained cable stress is limited to an allowable upper bound value; this criterion leads the determined cross-sectional area of each cable to fall within a feasible region (*T*_*i*_ ≤ *T*_allowable_).
(5) For the girders, the difference between the maximum positive and maximum negative moments should be limited to an acceptable range  (*M*_min⁡_ ≤ *M*_*j*_ ≤ *M*_max⁡_). This constraint introduces a local optimisation in which the corresponding cable force (*T*_*i*_) causing the moment of the girder to fall out of the acceptable range can be changed directly.

**Figure 2:**
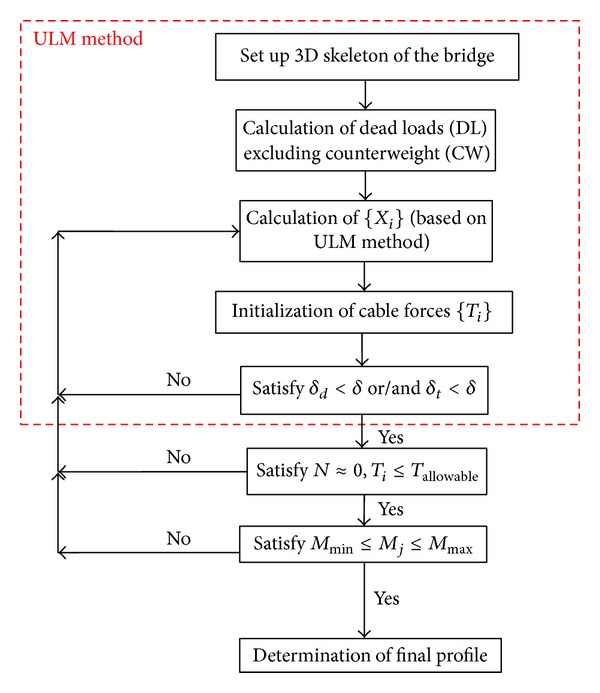
Scheme of multiconstraint method.

The scheme of proposed optimisation method is shown in Figure 2. Three iterative loops are applied to satisfy the constraints. The cable forces are firstly preestimated by applying the ULM and the deflection constraints (constraints (1) and (2) are checked). In the second stage, the axial stresses in the deck are checked (constraint (3)). Moreover, the pretension stress of each cable is controlled such that it does not exceed the allowable stress (constraint (4)). In the last stage, the bending moments of the girder are also checked to be in the considered range of uniformity (constraint (5)). If each of the introduced criteria was not satisfied, the procedure will iteratively be applied to satisfy all the constraints. As the proposed method is based on the ULM, the nonlinearities of the bridge are included, and it can be used in the construction process of highly redundant cable-stayed bridges.

## 3. Finite Element Modelling

**Figure 3:**
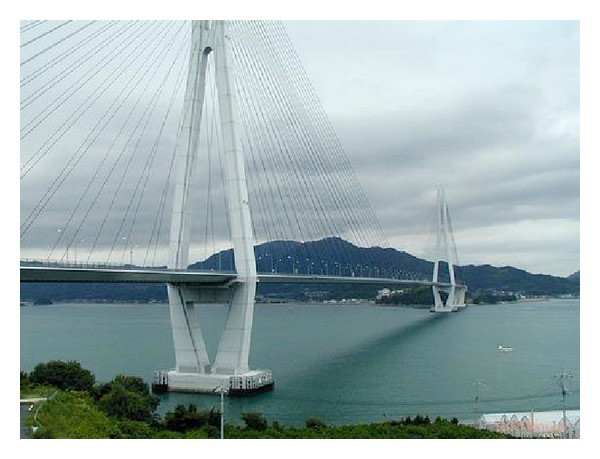
Tatara cable-stayed bridge.

The analytical model of Tatara bridge in Japan is considered for simulation in this study. The Tatara cable-stayed bridge, shown in Figure 3, has a total length of 1480 m, with a centre span of 890 m. The bridge has two lanes of traffic in each direction and additional lanes for bicycles, motorbikes, and pedestrians.

**Figure 4:**
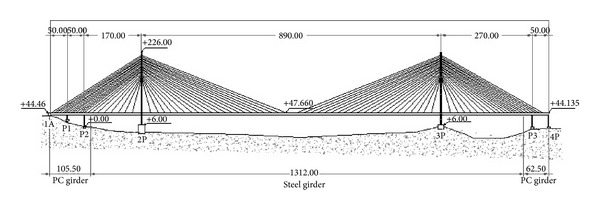
General arrangement of the Tatara cable-stayed bridge.

Figure 4 shows the general arrangement of the Tatara bridge. The main girder is a 3-cell steel box section in a streamlined shape that consists of three spans, which are 270 m, 890 m, and 320 m long, and 2.70 m deep. The suspended girders are streamlined steel box girders. The prestressed concrete (PC) girders are used as counterweight (CW) in the side spans to balance the weight of the main span. The inverted Y-shaped steel pylons with a height of 220 m have slits in the upper tower for aesthetic purposes and to enhance the aerodynamic effects of the structure. The stay cables are arranged in 21 levels and two planes with indented surfaces in the polyethylene cable coating to enhance their aerodynamic stability [22].

The FE analysis is used for simulation which is a prominent way for analysing cable-stayed bridges [23–27]. The geometric, inertial parameters, connections, and boundary conditions of the bridge are simulated in ANSYS software. The geometry and details of the model are based on the design data of the Tatara bridge. To reduce the degrees of freedom, a simplified three-dimensional FE model of the bridge is developed using elastic beam elements and link elements.

**Figure 5:**
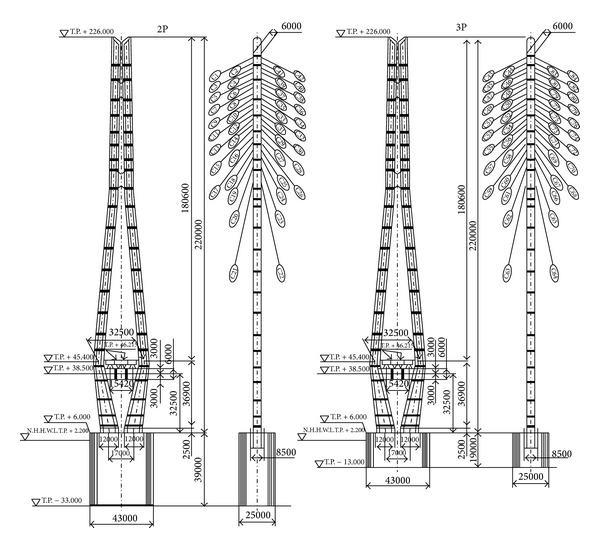
General arrangement of the main pylon of the Tatara bridge [22].

The bridge deck is modelled using a single central spine with offset rigid links to accommodate cable anchor points (fishbone model). The BEAM4 elements from the ANSYS element library are used to model the central spine. Stress-stiffening and large deflection capabilities are included in the model. The MPC184 elements are applied to model the rigid links, and MASS21 elements are used to include the mass of the equilibrium blocks, parapet, and anchors that are nonstructural members. The steel pylons, heads, and struts of the pylons are modelled as three-dimensional (3D) elastic BEAM4 elements. The general arrangement of the two pylons of the bridge is shown in Figure 5. The rigid links (MPC184 elements) are extended from the axial centre of the pylon to the cable anchor points. The detailed FE modelling procedure is discussed in the previous research works of authors [28, 29].

The cables of the bridge are parallel stranded cables with a tensile strength of 1,569 MPa. The cables are modelled in ANSYS by employing 3D nonlinear tension-only truss elements (LINK10) and utilising the stress-stiffening capability to consider the sag effect, which occurs because the cables do not have any bending stiffness [30]. The cables between the girder and the pylons are modelled as one-element cable system (OECS). Certain researchers have used multielement cable system (MECS) in simulation process [31–36]. However, simulating long-span cable-stayed bridges with MECS model demands considerably more computing time than OECS model [32]. As the main purpose of this study is studying the efficiency of the proposed optimization method, the OESC model is applied in this study with low computational costs. The OECS method has also been applied in the literature to optimize the cable forces of cable-stayed bridges [9, 10, 12, 37].

**Figure 6:**
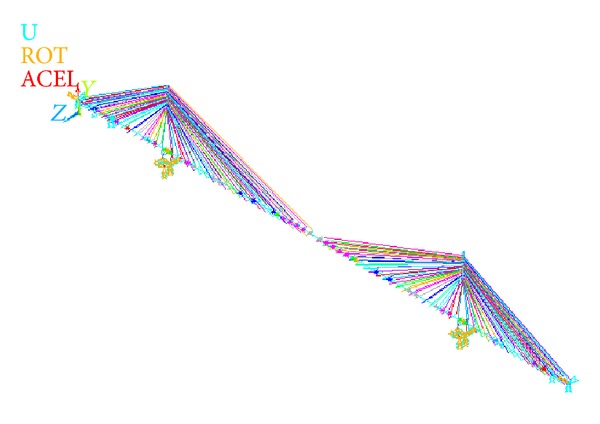
FE modelling of the bridge.

The boundary conditions at the piers, at the base of the pylons, and at the connection of each abutment are simulated in the FE model. The pylon bases are considered as being fixed in all degrees-of-freedom. The end connections permit the end of the deck to rotate freely about the vertical and transverse axes. Rotation about the longitudinal axis* (x)* and two translational degrees-of-freedom at each abutment are fixed. Elastic bearings were used for the pylon-to-deck connections. Thus, the spring constant about 3.92 × 10^6^ (N/m) is adopted to limit the girder displacement in the direction of the bridge axis based on design information about the bridge.  Figure 6 represents the FE modelling and boundary conditions assigned to the bridge.

### 3.1. Application of the Multiconstraint Method

The iterative multiconstraint method is applied in this study to find the pretensioning cable forces of simulated bridge. The cable forces of the bridge are preestimated by the ULM, utilising the deflection constraints (constraints (1) and (2) as mentioned in Section 2). To avoid convergence problems in the iterative procedure, the upper and lower bound of 2 × 10^−2^ m are considered to satisfy these constraints. However, the ULM does not produce well-distributed cable forces and girder moments in long-span cable-stayed bridges, as mentioned previously. The bending moments of the PC girders due to the dead load are extremely large compared with those of the steel girders, and the adjustment of these bending moments by cable prestressing alone is unrealistic. Furthermore, the effect of CWs on the final profile is not significant. Therefore, the weight of PC girders is excluded in the cable prestress determination.

Three more constraints are applied in the optimisation procedure in addition to the ULM deflection constraints to produce the optimum cable forces. The constraints are applied in 2 more steps (as shown in Figure 2) through an iterative solution. As constraint (3) would be easily satisfied after ULM optimisation (based on the analytical study results), constraints (3) and (4) (introduced in Section 2) are applied in one step to accelerate the iterative process. The iterations can be applied in most FE software packages. ANSYS is used in this study for the iterative optimisation process due to the capabilities of the software to solve iterative problems. The results of analytical study through iterative solution are presented as below for simulated cable-stayed bridge.

**Figure 7:**
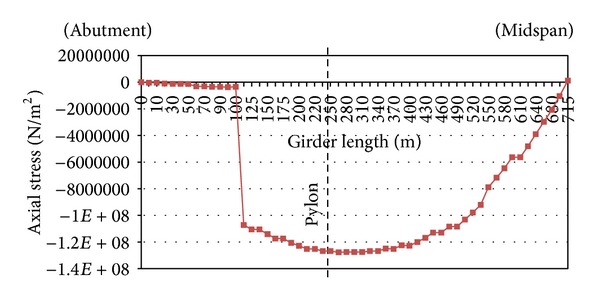
Axial stresses after the optimisation in the girder of the bridge.

The axial stress of the deck is checked through the iterative process.  Figure 7 indicates that the axial stress along the deck increases sharply at the connection of the PC girders to the steel girders and then grows slightly. As the PC girders are excluded from the process of finding pretensioning forces, it is reasonable that their axial stress from cable forces is negligible. From the pylon to the midspan, the stress decreases and almost reaches zero at the midspan of the deck. It can be found from Figure 7 that constraint (3) (as mentioned in Section 2) is completely satisfied through the optimisation method.

**Figure 8:**
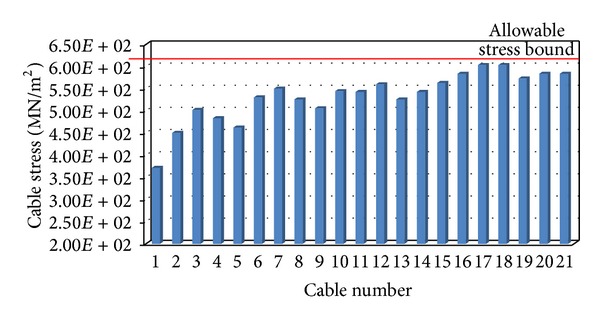
Distribution of the cable stresses after the optimisation.

As mentioned previously, the pretension stress of cables should not exceed the allowable stress of the cable (constraint (4) as mentioned in Section 2).  Figure 8 shows the pretensioning stresses in cables C1 to C21 after the optimisation and the allowable cable stress (the arrangement of cables C1 to C21 is shown in Figure 5). It can be found from Figure 8 that all the optimised pretensioning stresses are below the allowable range.  Figure 8 also indicates that cables C1 to C13 connected to CW girder have less pretensioning stresses because their dead load is excluded from the analysis, while the stress distribution in cables attached to main girder is in a certain level, resulting in an economical design.

**Figure 9:**
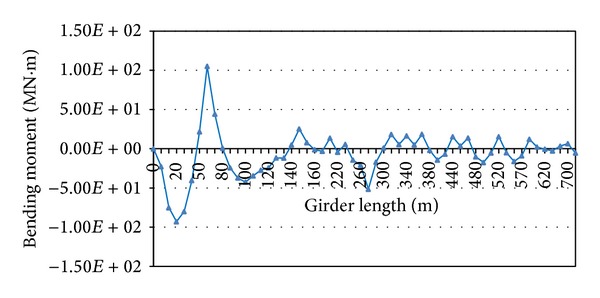
Distribution of the bending moments in the midspan of the bridge (multiconstraint method).

The bending moments of the deck are checked for uniformity (constraint (5) as mentioned in Section 2). Where the bending moment of each node of the steel girders is not in the considered range, the cable force is adjusted. The iteration continues until the optimum pretensioning cable forces are achieved. The range of 5 × 10^7^ N.m is considered for optimising bending moments in iterative process based on engineering judgement. However, the bending moment in PC girders and deck-to-pylon connections exceeds the considered range which is excluded from the iterative process manually to provide a uniform moment distribution in each span of the deck.  Figure 9 shows that the bending moments of the main girder are almost uniform after applying multiconstraint optimisation method. After the optimisation process, the bending moment along the pylons also decreases significantly. As mentioned previously, the bending moments in the PC girders are not adjusted by pretensioning cable forces; therefore, there are high bending moments in the PC girders as can be seen in Figure 9.

The analytical study shows that the optimization process may get locked in some local minimums. The solution for this problem is explained in Asgari et al. [38].

## 4. Comparative Study

**Figure 10:**
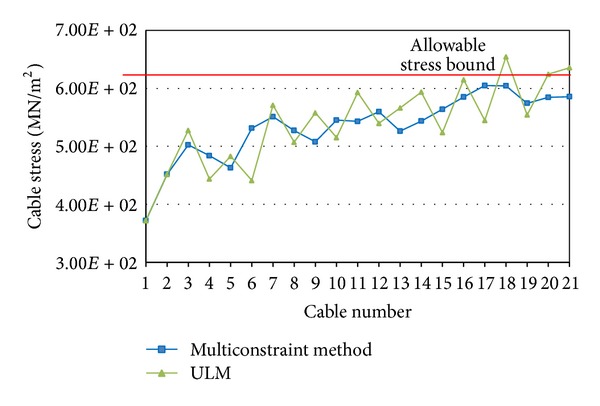
Comparison of pretensioning cable stresses.

A comparative study is conducted in this paper to compare the structural responses of the optimised cable-stayed bridge by multiconstraint method with responses derived from ULM.  Figure 10 shows the results of comparative study on the cable pretensioning stresses in cables C1 to C21 of the simulated bridge (the arrangement of cables C1 to C21 is shown in Figure 5).  Figure 10 illustrates that all the pretensioning stresses derived from multiconstraint method are below the allowable stress bound, while the optimised cable stresses by ULM in C18, C20, and C21 are higher than allowable stress.  Figure 10 also shows that the stress distribution is more uniform in multiconstraint optimisation method in comparison with ULM.

**Figure 11:**
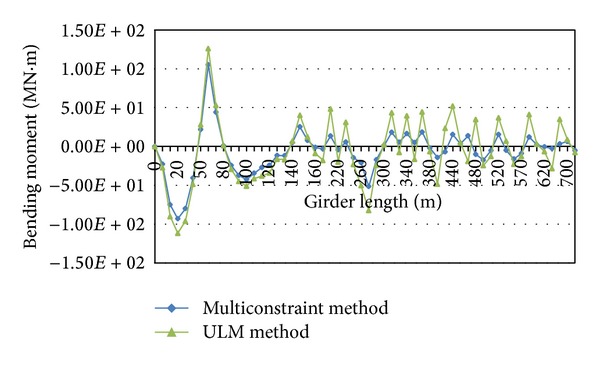
Distribution of the bending moments in the midspan of the bridge derived from multiconstraint method compared to ULM.

The comparison of the bending moment distributions in the simulated cable-stayed bridge (Figure 11) shows that the multiconstraint method is more successful than ULM method in providing uniform bending moments along the main girder of the bridge. This fact indicates that the multiconstraint method leads to more economical design than ULM.

The static and dynamic responses of the optimised cable-stayed bridge by multiconstraint method are also compared with the responses of ULM-optimised cable-stayed bridge to investigate the effect of pretensioning cable forces on the bridge responses. The results of static analysis shown in Table 1 indicate that the maximum bending moments at the pylon base and main girder decrease up to 53% and 39%, respectively, compared to the ULM. Furthermore, the deflection in the top point of pylon and deck midspan decreases by applying the proposed method. The decrease in the member stresses causes slender and lighter member sections, resulting in the optimum design of cable-stayed bridges.

The prestressed modal analysis is conducted after the static analysis to investigate the effect of the cable pretensions on the dynamic behaviour of the bridge. The modal analysis of the FE model starts from deformed equilibrium configuration of the bridge, which means prestressed modal analysis is conducted following the static analysis under the dead load and cable tensions to achieve the initial equilibrium configuration.  Table 2 shows the results of investigation on the natural frequencies of the prestressed modal analysis. As can be seen in Table 2, assigning the cable forces using the multiconstraint method increases the natural frequencies of the bridge slightly (less than 2%) comparing to ULM. It can be found from the results reported in Table 2 that the method of optimising cable forces does not have considerable effect on dynamic characteristics of the simulated cable-stayed bridge.

## 5. Conclusions

The determination of pretensioning cable forces is critical in the cable-stayed bridges design procedure. The results of investigations show that ULM method is a prominent method for optimizing the cable forces considering its easy extensibility, fast process, and applicability to real construction engineering problems. However, the cable forces optimised by ULM can be further improved because it does not produce well-distributed cable forces and girder moments in long-span cable-stayed bridges.

A new multiconstraint iterative algorithm is presented in this study to determine the cable pretensions of a long-span cable-stayed bridge, overcoming the limitations of previously proposed methods. The iterative procedure introduces three more constraints in addition to the conventional displacement constraints of the ULM. The additional constrains are assigned through 2 more iterative steps. As the proposed method follows ULM procedure in optimising cable forces, the time-dependent effects such as creep, shrinkage, or relaxations of pretensioning forces as well as geometrical nonlinearities can be considered. Furthermore, the proposed process can be used in the construction process of cable-stayed bridges.

The results of comparative study show that the proposed method further decreases the pylon base and main girder bending moments up to 53% and 39%, respectively, comparing to ULM. The maximum displacement at top point of the pylons and along girder also decreased which results in a safe design. Moreover, the multiconstraint method produces more uniform pretensioning forces in cables and also more uniform bending moment distribution along the deck, resulting in more economical design of the bridge. However, there is no significant difference between the dynamic characteristics of cable-stayed bridge optimised by ULM and multiconstraint method.

## Figures and Tables

**Table 1:** Static analysis results of the simulated cable-stayed bridge.

Analysis type	Maximum bending moment at pylon bases (N*·*m)	Maximum top pylon deflection (m)	Maximum bending moment in main girder (N*·*m)	Maximum girder deflection (m)
Static analysis with pretension in cables (ULM)	7.82 × 10^7^	0.07	7.93 × 10^6^	0.033
Static analysis with pretension in cables (multiconstraint method)	3.35 × 10^7^	0.009	4.81 × 10^6^	0.017

**Table 2:** Comparison of the natural frequencies (Hz).

Modes	ULM	Multiconstraint method
1st vertical	0.216	0.217
1st transverse	0.097	0.099
2nd transverse	0.242	0.243
2nd vertical	0.271	0.273
3rd vertical	0.361	0.362
1st torsion	0.437	0.439
3rd transverse	0.402	0.402
4th vertical	0.417	0.418
4th transverse	0.575	0.577
